# The Connection Between Social Inequality And Intergenerational Transfers Between Three Generations In Europe

**DOI:** 10.1093/geroni/igab046.2913

**Published:** 2021-12-17

**Authors:** Christian Deindl

**Affiliations:** TU Dortmund University, Dortmund, Nordrhein-Westfalen, Germany

## Abstract

Family members support each other across the entire family cycle. Parents help their adult children with financial transfers and hands-on-support and childcare, while children in mid-life often support their older parents with help and care. However, there is profound social inequalities linked to intergenerational transfers. While there is some research on inequality for some types of intergenerational transfers and some transfer directions, there is still no conclusive study bringing together all different support types between multiple generations from different social backgrounds over time. In our view, taking a longitudinal multi-generational perspective is essential to capture dependencies and negotiations within families from different socio-economic backgrounds within different regional contexts. If middle-aged parents have to take care of their own older parents, they have fewer resources for their(grand-)children, who might then receive less attention and support from them. This may differ according to access to support from public or private institutions. Here, country and regional specifics have a huge impact on support patterns within the family, which can only be captured when looking into developments and change. Using six waves of the Survey of Health, Ageing and Retirement in Europe (SHARE), we look at intergenerational transfers between multiple generations over time across European regions, considering mid-aged Europeans in the “sandwich” position between older parents and children and include multiple transfer directions and types over time to assess the links between social inequality and intergenerational solidarity in Europe’s ageing societies. The impact of Covid 19 on this issue will also be considered.

